# Gallbladder Agenesis: Report of a Preoperative Diagnosis With Magnetic Resonance Cholangiopancreatography

**DOI:** 10.7759/cureus.9647

**Published:** 2020-08-10

**Authors:** Beatrice D'Orazio, Fausto Famà, Guido Martorana, Gaetano Di Vita, Girolamo Geraci

**Affiliations:** 1 General Surgery, Department of Surgical Oncological and Stomatological Sciences, University of Palermo, Palermo, ITA; 2 Department of Human Pathology in Adulthood and Childhood "G. Barresi", University of Messina, Messina, ITA; 3 General and Oncological Surgery Unit, Fondazione G.Giglio, Cefalù, ITA; 4 Department of Surgical Oncological and Stomatological Sciences, University of Palermo, Palermo, ITA

**Keywords:** agenesis of gallbladder, conservative treatment, follow up

## Abstract

Agenesis of the gallbladder is an extremely rare congenital entity with shaded clinical and radiologic features, which make the preoperative diagnosis really challenging. Here, we report a case of a 52-year-old symptomatic female with biliary symptoms and contracted gallbladder at ultrasound (US). The final diagnosis was made with magnetic resonance cholangiopancreatography (MRCP) and the treatment was conservative. However, diagnosing this condition preoperatively is still challenging. However, with innovations in terms of biliary tract imaging technique, and with better knowledge of this entity, many unnecessary surgical procedures might be avoided.

## Introduction

Gallbladder agenesis (GA) is a rare disorder of the biliary tree (10-65 cases per 100,000) [1]. GA without extrahepatic biliary atresia (“isolated GA”) is a rare clinical congenital disorder (0.01%-0.06%, about 1 per 6500 live births), although the incidence is higher (up to 1%) in autoptic series [1-3]. The first reports of GA are dated between 1701 and 1702 by Lemery and Bergman and its pathogenesis is likely related to failure, during embryonic development, of the gallbladder and cystic duct to bud off from the common bile duct during the fifth week of gestation [3].

Young females are more commonly affected (3:1 ratio) and typically present the alteration in the second or third decade of life. Cases found in autopsies have an equal sex ratio [3].

Despite the absence of the gallbladder, half of the patients present with symptoms mimicking biliary colic, which is poorly understood [2-3].

Here, we present a case of a 52-year-old woman who presented with recurrent biliary colic and was diagnosed to have gallbladder agenesis on magnetic resonance cholangiopancreatography (MRCP).

## Case presentation

A 52-year-old woman presented as an outpatient after a post-prandial episode of right upper quadrant pain, dull, aching in quality, sudden in onset, colicky in nature, and radiated to the right scapula, with nausea and vomiting. These symptoms were worsened by meals and particularly by fatty food.

Her past medical and surgical history were unremarkable, with no history of use of tobacco, alcohol, or illicit drug reported.

Clinical family history was positive for cholecystectomies (father at the age of 45 due to gallstone disease and mother at the age of 39 for cholesterolosis).

The patient had exhibited a recent hepatobiliary ultrasound (US) as the initial workup for suspected gallbladder pathology with an 8 mm stone in her contracted gallbladder and was fasting, without signs of acute cholecystitis.

**Figure 1:**
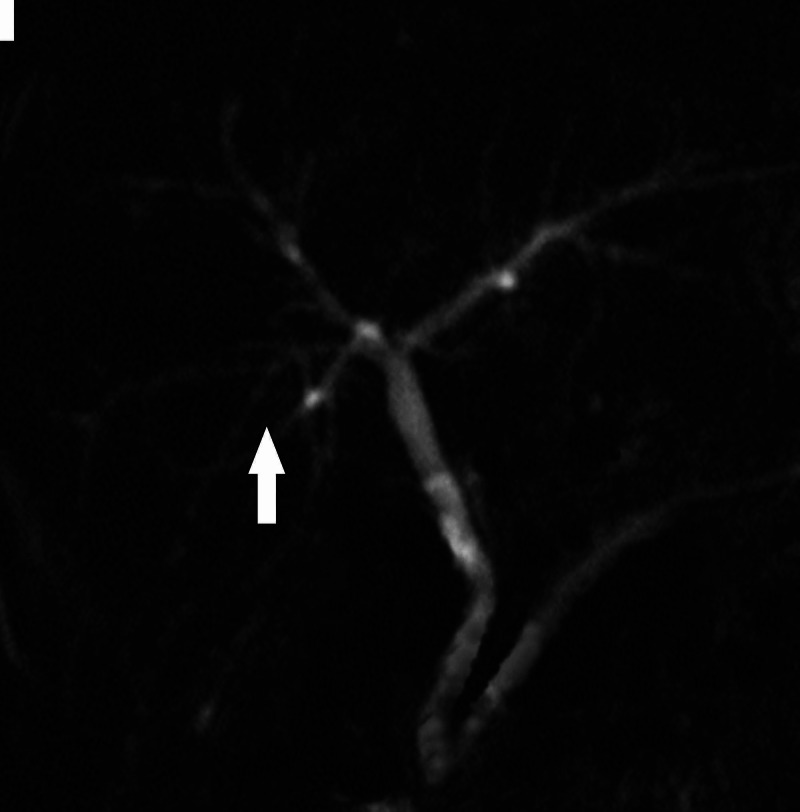
Magnetic resonance cholangiopancreatography (MRCP): image showing the absence of the gallbladder and the cystic duct, without any morphological alteration of the biliary tract.

Given the non-visualization of the gallbladder and on the basis of the clinical picture and further ultrasound, a computed tomography (CT) and a magnetic resonance cholangiopancreatography (MRCP) were prescribed: both tests did not show the gallbladder (compatible with post-cholecystectomy status); moreover, CT showed no other significant organ alterations and MRCP did not reveal any morphological alteration of the biliary tract (Figure 1).

At this point, a gastroenterology consult was called, for assistance with further nonsurgical management, with the diagnosis of agenesis of the gallbladder.

Although ERCP and sphincterotomy were considered as therapeutic alternatives, they were deferred as she responded well to medical treatment with 15 days of scopolamine-N-butilbromuro 10 mg per os twice a day (actually, hyoscyamine is not available in Italy).

Clinical and US follow-up at one year were uneventful.

## Discussion

For unclear reasons, despite the absence of a gallbladder, up to 50% of patients present with symptoms similar to biliary colic, probably because of an associated sphincter of Oddi dysfunction that may be the cause of biliary colic in these patients, or, in other cases, the development of common bile duct stones may be the cause [4]. As a matter of fact, our patient presented with symptoms mimicking biliary colic as for the timing and characteristics of the appearance and irradiation of pain.

GA would be found as an isolated entity in 70%-87.2% of cases of which 31.6% were asymptomatic and 55.6% symptomatic, while it occurs combined with other congenital alterations of the biliary tree in the remaining 12.8%-30% of cases [5].

GA is not associated with typical symptoms; as a matter of fact, approximately 23% of the patients present with symptoms mimicking a biliary condition occasionally during their lives, and bile duct stones might develop in 25%-60% of them [6].

This clinical presentation could be linked to a concomitant biliary disease, such as stones or dyskinesia, due to a significantly augmented sphincter of Oddi resting pressure, which leads to regurgitation of pancreatic or duodenal contents. It may also be caused by a condition unrelated to biliary tract diseases such as duodenitis, esophagitis, gastritis, or irritable bowel disease [7].

The best timing for the diagnosis of GA is preoperatively, but, unfortunately, this event has been reported only in few cases, as the US provides a sensitivity of <100% for the identification of the gall bladder and is highly operator-dependent [2].

Therefore, it is important to think of GA when the preoperative ultrasound is unable to visualize the gallbladder (hyperechoic material in the gallbladder fossa, small contracted, shrunken, scarred, sclerotic, or atrophied gallbladder); moreover, ultrasound is highly operator and habitus and bowel gas-dependent [1-2].

Clinicians should keep GA on their differential diagnosis list, and imaging modalities, such as MRCP, should be obtained when other tests prove inconclusive. In fact, MRCP is considered the test of choice if there is suspicion of GA, and it is also helpful in demonstrating other possible anomalies of the biliary tract [3,5,7].

New diagnostic techniques, such as biliary scintigraphy and hepatobiliary iminodiacetic acid (HIDA) scan, can potentially detect biliary anomalies and may be performed in some patients with gallbladder symptoms. In those patients, the non-visualization of the gall bladder may be attributed to cystic duct obstruction, so it may not be as informative as MRCP [3,5,8].

In our case, the patient had no other proven malformations of the biliary tree at CT scan or MRCP, while the pre-operative US suspected a calculous-contracted gallbladder.

Surgery can be risky in these patients because unnecessary dissection while looking for the non-existent gallbladder can result in an injury of the biliary tree, hepatic vasculature, or small bowel.

In the literature, there are no specific guidelines on how to manage GA, even if the use of hyoscyamine extended-release tablets twice daily may help to alleviate symptoms. The use of hyoscyamine in association with low-dose antidepressants, such as amitriptyline, can be useful to prevent any recurrence of symptoms, considering functional abdominal pain syndrome related to visceral hypersensitivity as a cause of unexplained biliary colic. No treatment or definitive procedure is required if no symptoms occur because the person with GA is healthy and the prognosis is excellent; regarding follow-up, although there are no guidelines, ultrasound control is considered sufficient for the evaluation of the dilation of the biliary tract and, possibly, an MRCP [9-11]. In light of our findings, we opted for a conservative medical therapy and one-year US follow-up.

## Conclusions

Preoperatively diagnosing gallbladder agenesis remains a challenge due to its rarity and shaded clinical and radiological features; nowadays, with the innovations in terms of imaging techniques, more and more cases are incidentally diagnosed. Nevertheless, it is mandatory to have a strong index of suspicion if non-visualization is suggested by an ultrasound. MRCP is considered the gold standard exam in case of clinical suspicion, as it provides accurate anatomic details of the biliary tree, preventing unnecessary surgery. The management, as in our case, is usually conservative, with smooth muscle relaxants.

## References

[1] Pipia I, Kenchadze G, Demetrashvili Z, Nemsadze G, Jamburia L, Zamtaradze T, Abiatari I (2018). Gallbladder agenesis: a case report and review of the literature. Int J Surg Case Rep.

[2] Naim H, Hasan SA, Khalid S, Abbass A, DSouza J (2017). Clinical cholecystitis in the absence of the gallbladder. Cureus.

[3] Tagliaferri E, Bergmann H, Hammans S, Shiraz A, Stuber E, Seidlmayer C (2016). Agenesis of gallbladder: role of clinical suspicion and magnetic resonance to avoid unnecessary surgery. Case Rep Gastroenterol.

[4] Salazar MC, Brownson KE, Nadzam GS, Duffy A, Roberts KE (2018). Gallbladder agenesis: a case report. Yale J Biol Med.

[5] Fiaschetti V, Calabrese G, Viarani S, Bazzocchi G, Simonetti G (2009). Gallbladder agenesis and cystic duct absence in an adult patient diagnosed by magnetic resonance cholangiography: report of a case and review of the literature. Case Rep Med.

[6] Cavazos-García R, Díaz-Elizondo JA, Flores-Villalba E, Rodríguez-García HA (2015). Gallbladder agenesis. Case report [Article in Spanish]. Cir Cir.

[7] Molnar C, Sárközi T, Kwizera C, Botoncea M, Zeno O, Petrișor M, Grigorescu BL (2019). Gallbladder agenesis. A rare congenital anomaly mimicking cholelithiasis in an adult woman [Article in Hungarian]. Orv Hetil.

[8] Arif SH, Mohammed AA (2020). Agenesis of the gallbladder, an unexpected finding during laparoscopy; case report. Ann Med Surg.

[9] Al-Hakkak SMM (2017). Agenesis of gallbladder in laparoscopic cholecystectomy—a case report. Int J Surg Case Rep.

[10] Rajkumar A, Piya A (2017). Gall bladder agenesis: a rare embryonic cause of recurrent biliary colic. Am J Case Rep.

[11] O'Sullivan J, O'Brien PA, MacFeely L, Whelton MJ (1987). Congenital absence of the gallbladder and cystic duct: nonoperative diagnosis. Am J Gastroenterol.

